# Stability and Biaxial Behavior of Fresh Cheese Coated with Nanoliposomes Encapsulating Grape Seed Tannins and Polysaccharides Using Immersion and Spray Methods

**DOI:** 10.3390/polym16111559

**Published:** 2024-05-31

**Authors:** Angela Monasterio, Emerson Núñez, Valeria Verdugo, Fernando A. Osorio

**Affiliations:** 1 Department of Food Science and Technology, Technological Faculty, University of Santiago-Chile (USACH), Av. El Belloto 3735, Estación Central, Santiago 9170022, Chile; angela.monasterio@usach.cl (A.M.); valeria.verdugo@usach.cl (V.V.); 2 Department of Fruit Production and Enology, School of Agricultural and Natural Systems, Pontificia Universidad Católica de Chile, Av. Vicuña Mackenna 4860, Macul, Santiago 7820436, Chile; ennunez@uc.cl

**Keywords:** biaxial viscosity, texture, cheese, nanoliposomes, tannins

## Abstract

In the food industry context, where fresh cheese stands out as a highly perishable product with a short shelf life, this study aimed to extend its preservation through multi-layer edible coatings. The overall objective was to analyze the biaxial behavior and texture of fresh cheese coated with nanoliposomes encapsulating grape seed tannins (NTs) and polysaccharides (hydroxypropyl methylcellulose; HPMC and kappa carrageenan; KC) using immersion and spray methods, establishing comparisons with uncoated cheeses and commercial samples, including an accelerated shelf-life study. NT, HPMC, and KC were employed as primary components in the multi-layer edible coatings, which were applied through immersion and spray. The results revealed significant improvements, such as a 20% reduction in weight loss and increased stability against oxidation, evidenced by a 30% lower peroxide index than the uncoated samples. These findings underscore the effectiveness of edible coatings in enhancing the quality and extending the shelf life of fresh cheese, highlighting the innovative application of nanoliposomes and polysaccharide blends and the relevance of applying this strategy in the food industry. In conclusion, this study provides a promising perspective for developing dairy products with improved properties, opening opportunities to meet market demands and enhance consumer acceptance.

## 1. Introduction

Fresh cheese, whether made from cow or goat milk, stands out as one of the highly perishable non-liquid foods, characterized by a short shelf life of 7 to 9 days [1,2] due to its high water and nutrient content leading to increased syneresis, decreased pH, and lipolysis [3]. The loss of quality, flavor, and texture indicates deterioration in cheeses, and this alteration can occur due to various factors, such as temperature and humidity, as well as internal factors inherent in the food composition [4]. One way to prevent food deterioration is by applying edible coatings, defined as a thin layer of edible materials applied directly to the surface of food, acting as a natural barrier against external conditions [5]. To address intrinsic factors affecting cheese deterioration, molecules with antioxidant and antimicrobial properties can be incorporated into edible coatings to enhance their functionality.

Condensed tannins or proanthocyanidins are phenolic compounds with high antioxidant capacity that provide various health benefits [6]. Techniques have been developed to incorporate these properties into different foods for therapeutic, structural, and preservative purposes. However, astringency and bitterness are the main organoleptic properties of these compounds [7]. The perception of these properties in the mouth is directly related to the ability of tannins to interact with salivary proteins, forming aggregates [8]; this makes their direct incorporation into foods challenging. Nanotechnology represents an innovative and versatile alternative to reduce unpleasant tastes in the mouth, produced either by the interaction between food components and saliva components or by the food’s nature [9,10].

Nanoliposomes are carrier systems for bioactive compounds widely used in various industries, such as cosmetics, pharmaceuticals, and food, as they exhibit excellent biocompatibility with biological membranes due to their amphiphilic nature [11]. Recently, lipid- and polysaccharide-based edible coatings have proven to be an effective strategy to improve product quality [12]. Biopolymers are considered non-toxic compounds, easy to modify and optimize, with some, such as hydroxypropyl methylcellulose and kappa carrageenan, providing structure to food matrices, thus improving their texture [13].

Coatings can be applied to or inside foods using various methods [14,15,16]. Immersion is the most common coating method for fresh products, ensuring good uniformity on complex and rough surfaces. It is also simple and cost-effective. On the other hand, spraying increases the liquid’s surface by forming small droplets and dispersing them through a set of nozzles onto the food surface [17,18].

The percentage increase in the weight of the samples coated by immersion was expressed in Equation (1).

(1) $$Weight\;gain\;\left(\%\right)=\frac{Initial\;weight-Final\;weight}{Initial\;weight}\times 100$$

For a steady nozzle centrifugal device, the atomization process, characterized by the mean drop diameter, D, is governed by the following physical parameters [19].

- *L*: The characteristic dimension of the atomizer, nozzle hole diameter.
- *U*: The initial relative velocity of the injected suspension and ambient air.
- *σ*: The surface tension of the coating.
- *ρ_s_*, *ρ_a_*: The densities of the coating suspension and air, respectively.
- *μ_s_*, *μ_a_*: The dynamic viscosities of the coating suspension and air.

After applying the Buckingham Pi theorem, the following relationship (Equation (2)) is obtained:

(2) $$\frac{D}{L}=f\left(R_{e},\;W_{e},\;\frac{\rho_{a}}{\rho_{s}},\frac{\mu_{a}}{\mu_{s}}\right)$$

With four dimensionless numbers, the first term corresponds to the Reynolds number, which represents the ratio of inertial force to viscous force:

(3) $$R_{e}=\frac{\rho UL}{\mu }$$

The second term represents the Weber number, which corresponds to the ratio of inertial force to surface tension force:

(4) $$W_{e}=\frac{U^{2}\rho L}{\sigma }$$

The third and fourth terms represent the density and viscosity ratios of the air and coating suspension used, respectively.

Combining the first two terms in Equation (2) to eliminate velocity allows obtaining another dimensionless number, the Ohnesorge number (*O_h_*), which highlights the relative importance of interfacial viscous and surface tension.

(5) $$O_{h}={W_{e}}^{0.5}{R_{e}}^{-1}=\frac{\mu }{{\left(\rho \sigma L\right)}^{0.5}}$$

Studies on determining texture in cheeses include lubricated uniaxial and biaxial compression tests, which determine extensional and compressional flows [20]. Young’s modulus and viscosity are essential parameters obtained through lubricated compression tests to define the texture of fresh cheeses (FCs) coagulated with commercial acids [21]. Lazárková et al. [22] evaluated the texture of white cheese in brine using stress–strain graphs corrected against Hencky deformation. Giliel et al. [23] used a double compression test to determine the hardness, elasticity, cohesion, and chewiness parameters in cheeses coated with a film-forming solution based on Ziziphus joazeiro fruit pulp.

Biaxial compression tests help determine the texture of hard, semi-hard, and soft cheeses. They are based on mechanical properties and conducted using specialized equipment. In these tests, the shape of the sample is maintained. At the same time, the contact area with the plate increases as the height of the sample decreases, thereby determining the cheese’s behavior in terms of stress and deformation [24,25].

The biaxial extension velocity distribution may be expressed in terms of the Hencky stress, ${\dot{\varepsilon }}_{h}$, as follows:

(6) $$u_{z}=-2{\dot{\varepsilon }}_{B}z={\dot{\varepsilon }}_{h}z$$

(6a) $$u_{r}={\dot{\varepsilon }}_{B}r=\frac{{\dot{\varepsilon }}_{h}r}{2}$$

(6b) $$u_{\theta }=0$$

where

$u_{z}$ = velocity in the z-direction [ms^−1^];
${\dot{\varepsilon }}_{B}={\dot{\varepsilon }}_{h}/2$ = biaxial extensional strain rate [s^−1^];
$u_{r}=$ velocity in the r-direction [ms^−1^];
*z* = axial coordinate [m];
${\dot{\varepsilon }}_{h}$ = Hencky strain rate [s^−1^];
$u_{\theta }=$ velocity in the θ-direction [ms^−1^].

The compression stress in the vertical direction is as follows:

(7) $$d{\dot{\varepsilon }}_{h}=-\frac{dh}{h}$$

where *h* = the height that separates the plates [m]. 

The biaxial extensional strain rate (also called radial extension rate) is equal to half of the Hencky vertical strain rate [24]:

(8) $${\dot{\varepsilon }}_{B}=\left(\frac{1}{2}\right){\dot{\varepsilon }}_{h}=\left(-\frac{1}{2h}\right)\frac{dh}{dt}=\frac{u_{z}}{2(ho-u_{z}t)}$$

where *h_o_* = initial height of the sample [m]; *h* = height [m]; *t* = time [s].

The extensional viscosity is calculated from the stretching stress and the strain rate.

(9) $$\eta_{B}=f\left(t\right)=\frac{\sigma_{B}}{{\dot{\varepsilon }}_{B}}=\frac{\sigma_{B}\;2(ho-u_{z}t)}{u_{z}}$$

where

$\eta_{B}=$ biaxial extensional viscosity [Pa s];
$\sigma_{B}$ = biaxial compression stress [Pa];
${\dot{\varepsilon }}_{B}$ = biaxial extensional strain rate.

The biaxial compression stress ($\sigma_{B}$) is obtained from the following expression:

(10) $$\sigma_{B}=\frac{Fh}{\pi {R_{o}}^{2}ho}$$

where

*R_o_* = initial radius of the sample [m];
*F* = force [N].

Moreover, and as a complement to the mentioned studies, the composition of the food is a relevant factor for result analysis, as the content of proteins, fat, water, and carbohydrates affects the rheological behavior and, therefore, the textural characteristics [26]. In this context, the objective of this research was to analyze the biaxial behavior and texture of fresh cheese coated with nanoliposomes encapsulating grape seed tannins (NTs) and polysaccharides (HPMC and KC) using immersion and spray methods to establish comparisons with uncoated cheeses and commercial samples, including an accelerated shelf-life study.

Studying texture in coated cheeses is fundamental as it is considered a quality attribute that influences sensory perception, consumer acceptability, and the cheeses’ characterization, classification, and processing. Moreover, it provides valuable information for manufacturers and consumers and contributes to the continuous improvement of dairy products. The potential benefits associated with the research encompass a general improvement in product quality, strengthening its structure, and adding antioxidant capacity to the matrix, leading to new products, improvements in food processing, and product shelf life.

## 2. Materials and Methods

### 2.1. Materials

Condensed tannin powder with a medium degree of polymerization of 2.5 ± 0.2, a galloylation degree of 15.5 ± 1.1, and an average molecular weight of 784 ± 61 was used, sourced from the Enology Laboratory at the Pontifical Catholic University of Chile. Dimerco Comercial Ltd. provided soy lecithin (Santiago, Chile). Glycerol PA (purity >99%) and sodium thiosulfate were purchased from Sigma Aldrich (St. Louis, MO, USA). Ethanol PA from Merck, HPLC-grade methanol (≥99.9%), Trifluoroacetic acid (TFA) from Merck (Darmstadt, Germany), and Milli-Q water were used as solvents. Kappa carrageenan (Carragel PGU 5289, Gelymar^®^, Santiago, Chile) and HPMC (Methocel E19, Dow Wolff Cellulosics, Bomlitz, Germany) were employed. The liquid rennet CHY-MAX^®^ M200 for FC preparation was obtained from DILACO (Santiago, Chile).

### 2.2. Experimental Design

To determine the optimal concentrations of Tween-80 and GLY to be added in the preparation of nanoliposomes encapsulating tannins (NTs), a multi-level factorial design with 18 experimental runs was conducted in two blocks [27]. The factors, levels, and evaluated response variables are presented in Table 1.

In this experimental design, three levels were considered for the concentrations of each factor. The evaluated response variables were density, surface tension, and optical density. The design matrix was generated using the STATGRAPHICS Centurion XVI software (v. 16.1.03), and the experiments were conducted in random order to minimize bias. Subsequently, multiple optimizations of the response variables were performed using the desirability function, where all the variables were maximized. This approach aimed to enhance the encapsulating material per unit volume, potentially improving the retention capacity of active compounds. Simultaneously, higher surface tension leads to the formation of smaller droplets, favoring the uniform dispersion of coating components [28].

Additionally, Tween-80 and GLY concentrations were selected based on their critical roles in emulsion stability and nanoliposome formation. The decision to focus on these two factors was supported by previous research indicating their significant impact on nanoliposome properties [29]. While other formulation components were not included in the factorial design, their levels were kept constant based on preliminary experiments and literature review. This ensured a focused investigation of the main factors affecting nanoliposome characteristics [30]. This approach allowed for a more efficient exploration of the key parameters influencing the encapsulation process.

### 2.3. Nanoliposomes Encapsulating Tannins (NTs)

The formation of NT is based on the methodology described by Jafari et al. [31] with some specific modifications. An oily phase was prepared, composed of Tween-80, grape seed oil (OG), GLY, and Milli-Q water. Simultaneously, an aqueous phase containing tannins in suspension (TS) in citrate buffer (0.1 M at pH 3) was prepared. Subsequently, the aqueous phase was gradually incorporated into the oily phase using a programmable syringe pump to control the flow and prevent the formation of aggregates. After incorporation, the mixture was homogenized using an Ultraturrax (IKA T18, Staufen, Germany) at 10,000 rpm for 3 min. Then, it underwent 5 min of sonication to obtain liposomes using an ultrasonic cell disruptor (HIELSCHER UP100H, Teltow, Germany, max. 100 W) with an MS7 Micro tip 7 sonotrode (7 mm in diameter, 120 mm in length, 130 W/cm^2^ acoustic power density) working at 50% amplitude. Empty nanoliposomes (ENs) were used as a control. The resulting liposomal suspension was stored at 4 °C to preserve its stability, and finally, the efficiency of encapsulation and stability analyses were conducted before its use as the main ingredient in multilayer edible coatings (MECs).

### 2.4. Encapsulation Efficiency (EE)

The methodology described by Babazadeh et al. [32] was employed to determine EE. Two milliliters (2 mL) of NT were centrifuged at 4 °C and 14,000 rpm for 1 h (Hanil Scientific Inc. Supra R22, Gimpo, Republic of Korea) to separate the suspended tannins. The supernatant was filtered through 0.22 μm syringe filters and deposited in borosilicate vials for analysis via UHPLC (Thermo Scientific Dionex UltiMate 3000, Waltham, MA, USA). A C18 column (5 μm, 250 × 4.6 mm, Perkin Elmer, Shelton, CT, USA) and a UV detector were used for tannin analysis. The mobile phases and gradients employed were as described by Bianchi et al. [33]. Epicatechin monomer was used as the standard for tannin quantification, with a calibration curve (5–500 μg/mL, R^2^ = 0.999). Epicatechin detection was performed at 280 nm. The EE in percentage was determined in triplicate and calculated using Equation (11).

(11) $$EE=\frac{\left[TE]-[TL\right]}{\left[TE\right]}\cdot 100$$

where

[*TE*]: The initial concentration of encapsulated tannins, [mg of epicatechin/g of sample].
[*TL*]: The concentration of free tannins in suspension, [mg of epicatechin/g of sample].

### 2.5. Stability Study of NT

The stability study of NT was conducted using a LITESIZER 500 particle analyzer (Anton Paar, Graz, Austria). The analysis included determining the particle size, polydispersity index, diffusion coefficient, zeta potential, conductivity, and transmittance for each sample over a 15-day storage period at 4 °C. A backscatter measurement angle of 175° was employed [34,35].

### 2.6. Film-Forming Suspensions Based on Polysaccharides (FSs)

HPMC and KC were used as polysaccharides to prepare film-forming suspensions. Milli-Q water was heated to the dissolution temperature of each compound and stirred magnetically at 1400 rpm for 15 min. After this time, the polysaccharide was slowly and gradually added in a thin stream until complete dissolution. Once the polymer was dissolved, it was tempered to 20 °C and dispersed using an Ultraturrax at 10,000 rpm for 5 min. The resulting film-forming suspension was degassed in an ultrasound bath and stored under refrigeration [36].

### 2.7. Formation of Multilayer Edible Coatings (MECs)

In this section, we employed the experimental designs previously conducted by our research group to systematically vary the concentrations of components and optimize relevant variables for creating multilayer edible coatings (MECs).

The selected NT from the experimental design and the FS-HPMC and FS-KC were used for the formation of the MEC. The first coating comprised 65% NT and 35% HPMC, designated as coating A. The second coating was prepared with 75% NT and 25% KC, designated as coating B. Both A and B were prepared using magnetic stirring at 1400 rpm and 20 °C for 1 h. After this time, the mixture was homogenized using an Ultraturrax at 3000 rpm for 15 s [37].

### 2.8. Preparation of Fresh Cheese

The methodology described by Nemati et al. [38] was employed to produce fresh cheese with some modifications. Semi-skimmed cow’s milk was pasteurized at 75 °C for 15 s. After this thermal process, the milk was cooled to 35 ± 2 °C, and CHY-MAX^®^ M200 liquid rennet, derived from Aspergillus niger, and calcium chloride were added. This inoculation was allowed to rest for 30 min. Afterwards, the coagulum was cut into approximately 2 cm cubes, and sodium chloride was added to salt the mixture. The draining and molding process took place in a cheese vat measuring 10 × 10 cm^2^, with weight applied, at refrigeration temperature for 24 h.

The commercial samples of goat cheese (GC) and vegan cheese substitute (VC), used for texture and biaxial behavior comparisons, were purchased from a local supermarket in Estación Central, Santiago, Chile.

### 2.9. Comparison with Commercial Samples

This study compares our experimental cheese samples with the commercial samples of goat cheese and a vegan cheese substitute. This comparison highlights nanoliposome coatings’ potential advantages and unique properties on the experimental samples. While it is acknowledged that the commercial samples differ significantly in composition and were not coated with liposomes, this comparison is still valid and provides meaningful insights for several reasons.

Firstly, commercial goat cheese and vegan cheese substitutes represent standard benchmarks in the market. By comparing our experimental samples to these established products, we can evaluate how the liposome coatings influence key properties such as texture, shelf life, and nutritional content relative to products consumers are already familiar with. This helps contextualize the experimental cheese’s performance within the broader market.

Secondly, although the commercial samples were not coated with liposomes, the comparison allows us to identify specific areas where the liposome coatings offer clear benefits. For example, improvements in the stability and controlled release of bioactive compounds in the experimental samples can be directly contrasted with the properties of the commercial products; this helps demonstrate the added value liposome technology can bring to cheese production.

Finally, the study aims to pave the way for future research and development. Including the commercial samples without liposome coatings provides a baseline against which future studies could compare results when similar coatings are applied to commercial products. Future research could involve applying nanoliposome coatings to commercial cheeses and vegan substitutes, allowing for a more direct comparison and validation of the observed benefits.

### 2.10. Proximate Composition

The proximate analysis of the cheeses included the determination of moisture content through the gravimetric method described in A.O.A.C. 925.45 standard, protein content using the Kjeldahl method as proposed by A.O.A.C. 990.03 standard, lipid or fat content through the alkaline hydrolysis methodology described in A.O.A.C. 996.06 standard, and the total ash content of the samples determined in a muffle furnace at 500 °C according to A.O.A.C. 923.03 standard [39,40,41,42]. Carbohydrates were calculated by difference from the previously obtained data and expressed as a percentage [43].

### 2.11. Dipping Coating Process

Pieces of fresh cheese were immersed in the coating suspension for 5 s to apply the MEC to the samples. Subsequently, the coated cheeses were placed on mesh screens to allow drying at room temperature (20 °C) for 1 h. Afterwards, they were transferred to individual aluminum trays with perforations and stored under refrigeration conditions at a constant temperature of 4 °C for six days, following the procedure outlined by Vasiliauskaite et al. [44].

### 2.12. Spray Coating Process

In forming an edible layer on the surface of fresh cheese, an experimental approach based on a pilot spray system was followed, as previously described by Silva-Vera et al. [45]. The liquid flow was controlled using a rotameter connected to the supply line to carry out this task. The MEC needed for layer formation was prepared in a sealed 2.5-L tank. A spraying device from Spraying System S.S. Co (model VA67255–60° S.S., Glendale Heights, IL, USA) and air atomizing caps from the same brand (model VF2850–SS) were used to optimize the process. The experimental tests were conducted at 5 [L/h], 50 [kPa] pressure, and 0.3 [m] height. This combination of variables was applied to the cheese samples for 20 s. After application, the samples were initially stored at room temperature (20 °C) for 1 h for the formation and setting of the edible layer, followed by storage under refrigeration conditions (4° C).

### 2.13. Lubricated Compression Test

A lubricated compression test was conducted to determine the biaxial behavior of the samples to minimize friction and ensure extensional deformation. Rectangular prisms of cheese measuring 5 cm in length × 2 cm in width × 1.5 cm in height were cut and positioned between two parallel plates lubricated with vegetable oil, attached to a universal testing machine (Zwick/Roell BDO-FBO.5T5 Texture Analyzer, Ulm, Germany). The samples underwent biaxial deformation at a constant deformation rate (1 mm/s) and were compressed to 85% of their initial height. The obtained data files were transferred to Microsoft Excel^®^ (v.2404/2019) for subsequent curve fitting [46].

### 2.14. Weight Loss (WL)

The percentage weight loss of the cheeses was determined by subtracting the initial weight of the cheese from the weights measured at various intervals during storage under accelerated conditions (30 °C), as per Equation (12) [47].

(12) $$WL=\frac{\left[W_{i}]-[W_{t}\right]}{\left[W_{i}\right]}\cdot 100$$

where

$W_{i}$: The initial weight of the cheese.
$W_{t}$: The weight of the cheese at time t.

### 2.15. Peroxide Index (PI)

The cheese samples with coatings were subjected to accelerated storage conditions at 30 °C for 6 days, following the methodology described by Sánchez-González and Pérez [48]. The peroxide index was determined according to the UNE-ISO 3960-2017 [49] standard for oils and fats of animal and vegetable origin. A 0.3 [g] sample was weighed and dissolved in 30 [mL] of acetic acid–chloroform solution (3:2). Subsequently, 3 [g] of potassium iodide and 500 [µL] of distilled water were added, and the mixture was stirred for 1 min. Then, 30 [mL] of distilled water and 1.5 [mL] of a 1% starch solution were incorporated as an indicator. Titration was carried out with 0.001 N sodium thiosulfate until a color change was observed. Blank titration was performed, and the peroxide index was calculated according to Equation (13).

(13) $$PI=\frac{\left(V_{M}-V_{B}\right)\cdot N\cdot 1000}{m}$$

where

*PI*: Peroxide index expressed in Meq. O_2_/kg of cheese.
$V_{M}$: The volume of sodium thiosulfate spent on the sample.
$V_{B}$: The volume of sodium thiosulfate spent on the blank.
*N*: The normality of sodium thiosulfate.
*m*: The mass of cheese used.

### 2.16. Statistical Analysis

All the assays outlined in this manuscript were conducted in triplicate, and the experimental data obtained were expressed as mean ± standard deviation. Differences among three or more groups were assessed using ANOVA tests, followed by Tukey’s comparison tests with a confidence level of 95% (*p* < 0.05) to establish statistical significance [50]. All the statistical analyses were performed using the STATGRAPHICS Centurion XVI software, v.16.1.03 (StatPoint Technologies, Inc., Warrenton, VA, USA).

## 3. Results

### 3.1. Multi-Level Factorial Design

**Figure 1:**
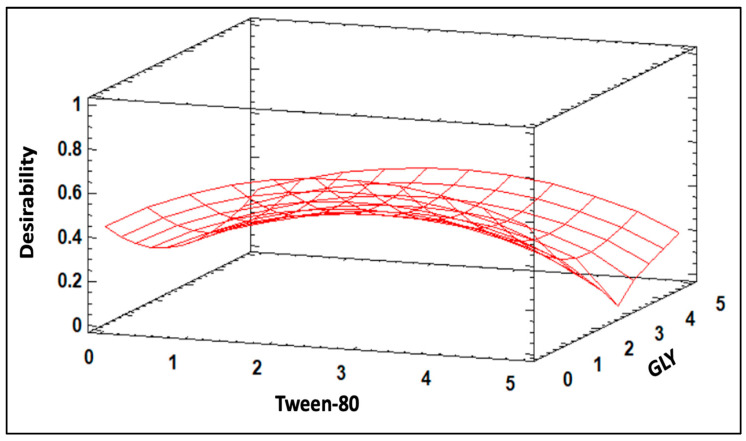
Estimated response surface for NT ingredients.

During our research, we implemented a multi-level factorial design to optimize nanoliposome composition (NT) for grape seed oil formulation. Figure 1 displays the estimated response surface detailing the optimal combination of Tween-80 and GLY. The results revealed that the optimal concentration of Tween-80 was 2.6%, while that of GLY was 1% relative to the amount of grape seed oil. These proportions were critical for maximizing the nanoliposome’s efficacy and stability. Under these optimal conditions, a desirability value of 0.55 was attained, indicating an ideal combination that maximizes the nanoliposome’s desired properties. These findings underscore the significance of meticulous component optimization in nanoliposome formulation, emphasizing efficiency in encapsulating bioactive compounds and ensuring the quality and stability of the final product.

### 3.2. Encapsulation Efficiency (EE)

Within the scope of our research, we assessed the EE for the epicatechin monomer in NT derived from grape seed tannin powder, Tween-80, and glycerol. The results unveil a significant EE, registering at 76 ± 0.03 [%]. This metric attests to the effectiveness of the encapsulation process, showcasing the nanoliposomes’ capability to retain and safeguard the epicatechin monomer during formulation.

The notable 76% EE underscores the efficiency of the specific combination of the components employed in the nanoliposome synthesis. As determined in the earlier multi-level factorial design, the presence of Tween-80 and glycerol optimizes the desirability function and contributes significantly to the encapsulation efficiency. The nanoliposomes’ ability to encapsulate the epicatechin monomer with such efficacy is pivotal for ensuring the stability and bioavailability of the compound across diverse applications, spanning from the food industry to pharmaceuticals.

These findings reinforce the relevance of precise formulation in nanoliposome synthesis for the efficient encapsulation of bioactive compounds, thus promoting their successful application across various scientific and industrial domains.

### 3.3. Stability Study of NT

Table 2 shows the comprehensive results of a stability study conducted on nanoliposomes (NTs) throughout a 15-day refrigerated storage period. The mean particle size (MPS), polydispersity index (PDI), diffusion coefficient, Z-potential, conductivity, and transmittance parameters play crucial roles in determining the stability and behavior of the nanoparticles in storage conditions. The results provide valuable insights into the dynamic evolution of NT characteristics over time, shedding light on their potential applications in diverse fields.

The data indicate that nanoliposomes exhibit significant stability over the 15 days, with only minor fluctuations in critical parameters such as MPS, PDI, and Z-potential. This stability is crucial for their potential use in various industries. For instance, in the pharmaceutical industry, stable nanoliposomes can be used as drug delivery systems, where consistent particle size and charge are essential for the effective and controlled release of therapeutic agents [51]. The cosmetic industry can also benefit from these findings, as stable nanoliposomes can enhance the delivery of active ingredients in skincare products, improving their efficacy and shelf life [52]. Additionally, in the food industry, nanoliposome stability can be leveraged to encapsulate and protect sensitive bioactive compounds, such as vitamins and antioxidants, ensuring their sustained release and enhancing the nutritional profile of food products [53].

These applications underscore the importance of understanding and optimizing nanoliposome stability under various storage conditions. Future research could explore the long-term stability of these nanocarriers and their behavior under different environmental stresses, further broadening their potential industrial applications.

### 3.4. Proximate Analysis

Table 3 presents the proximate composition of the FC coated with MEC based on NT and polysaccharides such as HPMC and KC, alongside the commercial GC and VC samples. Significant variations in moisture, proteins, lipids, ashes, and carbohydrates are observed among the samples, indicating the coatings’ differential impact on the cheeses’ nutritional composition. These results provide a crucial starting point for the detailed discussion on how the properties of the coatings, especially those incorporating nanoliposomes, influence the composition and characteristics of fresh cheeses and their relevance in comparison to commercial and vegan products.

### 3.5. Biaxial Behavior of Cheeses Coated with MEC

**Figure 2:**
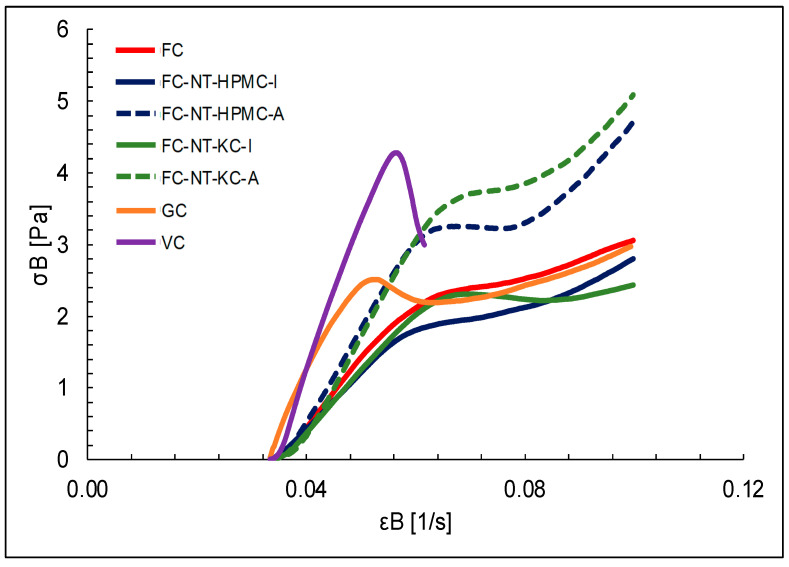
Biaxial stress rate versus biaxial extensional strain rate for different types of cheese.

**Figure 3:**
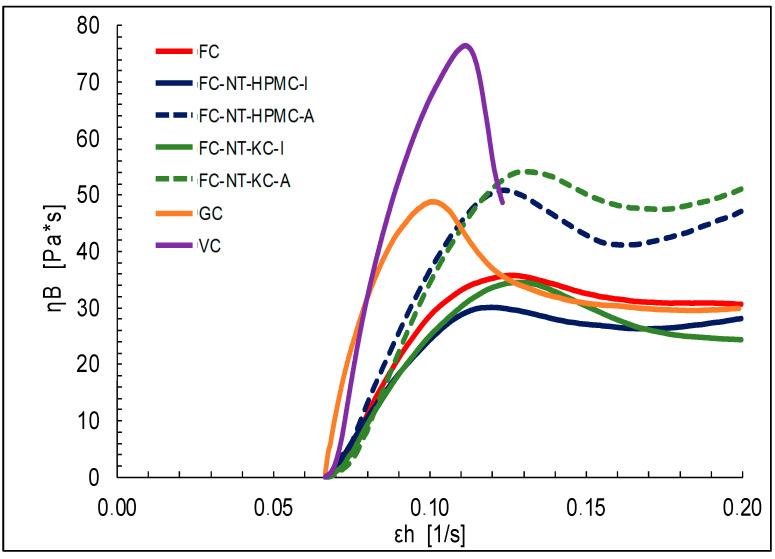
Biaxial extensional viscosity versus Hencky strain rate for different types of cheese.

Figure 2 and Figure 3 depict the results of the biaxial behavior of the cheese samples, derived from the Hencky stress–strain equations when a compressive force is applied in the vertical direction.

The obtained data provide a detailed insight into how various factors influence the texture of the cheese samples subjected to biaxial compression. Firstly, the influence of the coating type becomes apparent, with the NT-HPMC-coated samples requiring a higher force for deformation compared to those coated with NT-KC.

**Figure 4:**
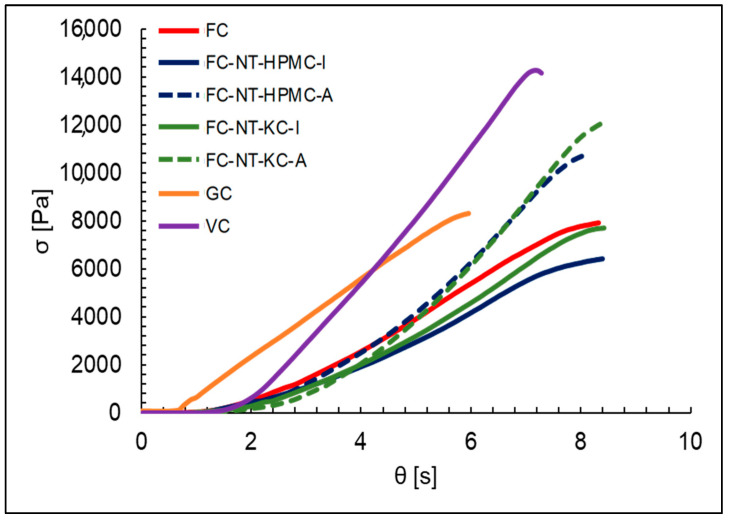
Modeling of the biaxial behavior for different types of cheese at a compression speed of 1 mm/s.

To model biaxial behavior, a linear fit was applied to the compressive stress curves [Pa] against time *θ* [s], represented in Equations (14) and (15), where *θ* represents the difference between the compression time and the stabilization of the test. This time adjustment corresponds to the difference between the actual compression time (*t*) and, as visualized in Figure 4, an initial time threshold (*t*_0_) passing through the origin.

(14) σ=aθ

(15) $$\sigma =a(t-t_{0})$$

where

σ: Compressive stress [Pa].
a: The slope of the line [Pa/s].
θ = $\left(t-t_{0}\right)$: Difference between the compression time and the initial time threshold [s].

The results of fitting the biaxial behavior curves for different cheese types at a compression rate of 1 mm/s are summarized in Table 4.

### 3.6. Quality Parameters in Fresh Cheese

**Figure 5:**
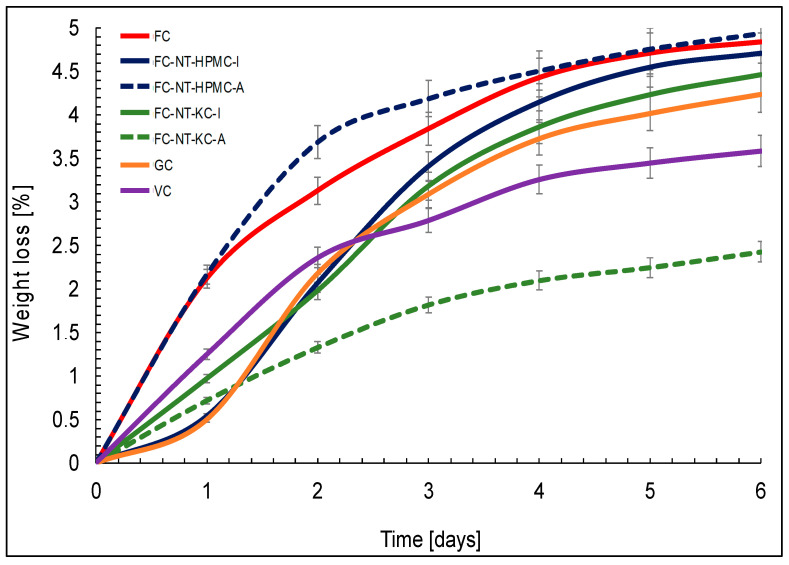
Weight loss for samples of cheeses coated with MEC and commercial cheeses versus storage time under accelerated conditions.

Figure 5 reveals information on weight loss in the different cheese samples over six-day storage under accelerated conditions. The FC sample showed a gradual weight loss, which is expected due to natural dehydration during ripening. The low weight loss value suggests that the uncoated FC maintains predictable weight loss over time.

Furthermore, the peroxide index indicates the amount of peroxides (oxidizing compounds) present in a sample. It is commonly used as an indicator of the rancidity or oxidation of a product. The results presented in Table 5 provide a detailed insight into how the peroxide index evolves in the different cheese samples over a six-day storage period under accelerated conditions.

## 4. Discussion

### 4.1. Multi-Level Factorial Design 

The observed outcome underscores the pivotal roles played by both Tween-80 and GLY in optimizing the desirability function value. Glycerol, known for its humectant and plasticizing properties, emerges as a critical component [54]. Its inclusion enhances the film-forming capacity and texture of the MEC. Glycerol’s humectant properties contribute to moisture retention, a feature particularly beneficial for preserving freshness in cheese products. Moreover, its plasticizing characteristics augment the overall flexibility and pliability of the MEC, contributing to improved film integrity. These attributes are of paramount importance for the successful application of MEC in fresh cheese production, where factors such as texture, film-forming capacity, and moisture retention play crucial roles in determining the quality and shelf life of the product. Thus, the synergy between Tween-80 and glycerol emerges as a strategic formulation approach, optimizing both functional and organoleptic attributes for enhanced applicability in fresh cheese manufacturing.

### 4.2. Encapsulation Efficiency (EE)

The observed enhancement in EE with the application of ultrasound suggests a notable facilitation of the encapsulation process for smaller units of condensed tannins, as highlighted by the findings of Rosales et al. [55]. Ultrasound, known for its ability to induce controlled cavitation and enhance mass transfer, likely plays a pivotal role in promoting the encapsulation of these smaller tannin units, resulting in the heightened efficiency observed in our study.

Furthermore, the influence of citric acid, a tricarboxylic acid, on encapsulation is noteworthy. As a crystalline solid under normal conditions, citric acid’s high solubility in water becomes a critical factor in forming encapsulation complexes with epicatechin compounds during the NT fabrication process, as discussed by Munin et al. [56]. The hydroxyl group’s positioning at position 3 in epicatechin introduces subtle molecular interactions within the encapsulation matrix. These interactions, along with the three-dimensional structure of the molecule, potentially contribute to variations in EE, as proposed by Li et al. [57].

Regarding the interaction between lipidic particles (NTs) and carbohydrate polymers, it is essential to acknowledge that these interactions can be complex and somewhat unpredictable due to the heterogeneous nature of the nanoliposome matrix. However, these intricate interactions may play a crucial role in imparting stability to the coating.

The reported EE values align with prior studies demonstrating high EE for nanoliposomes loaded with diverse bioactive compounds, such as alpha-linolenic acid (ALA), an omega-3-rich fatty acid, and polyphenols like naringin and naringenin [58,59,60]. The consistency in EE values across different studies underscores the robustness of the nanoliposome platform for encapsulating a broad range of bioactive compounds, showcasing its potential for diverse applications in the delivery of therapeutic and nutritional agents.

### 4.3. Stability Study of NT

The stability study of NT revealed several dynamic interactions between its components, shedding light on their role in maintaining the nanoliposome’s stability. The observed fluctuations in key parameters provide valuable insights into the complex interplay of factors influencing nanoliposome behavior during refrigerated storage.

The mean particle size (MPS) fluctuations observed in the initial days, followed by stabilization around 331 ± 5 [nm] by day 6 (as shown in Table 2), may be indicative of an equilibrium point reached in the aggregation–disaggregation processes of the nanoparticles [61]. The dynamic nature of nanoliposome interactions can result in variations in MPS, and the observed stabilization could suggest a balance between aggregation and disaggregation phenomena [62].

Furthermore, the interactions between lipidic particles (NTs) and carbohydrate polymers, such as HPMC and KC, which constitute the multilayer edible coatings (MECs), play a crucial role in coated nanoliposome stability. The hydrophobic–hydrophilic balance between the lipidic particles and the polysaccharide matrix influences the nanoliposome’s overall stability by affecting their aggregation–disaggregation dynamics and surface charge properties [63].

The polydispersity index (PDI) values, consistently within a moderate range, indicate the relatively uniform dispersion of particle sizes. The stability in PDI implies that the nanoliposomes maintained a relatively homogenous size distribution over the refrigerated storage period [64].

The peak in the diffusion coefficient on day 2, indicating enhanced particle movement or diffusion, provides valuable insights into the mobility of nanoliposomes. This understanding can significantly impact their behavior in various applications such as drug delivery or catalysis [65].

The Z-potential values, representing the surface charge of NT, declined until day eight before stabilizing. Changes in Z-potential can influence the stability of colloidal systems, and the observed variations suggest alterations in the electrostatic interactions governing nanoliposome stability [66]. Further investigations into the factors influencing surface charge dynamics could enhance our understanding of nanoliposome behavior.

The conductivity fluctuations, which reached a minimum on day 7, could potentially indicate alterations in the ionic strength or conductivity of the surrounding media. These variations might be attributed to changes in the nanoliposome’s environment, such as interactions with the storage medium or potential surface modifications [67].

The transmittance values remained stable, indicating the maintenance of optical clarity [68]; this is crucial for applications involving transparent or translucent materials, and the consistent transmittance values suggest the preservation of the NT optical properties over the storage period.

These results suggest a dynamic interplay of factors influencing nanoliposome stability during refrigerated storage. The observed fluctuations in key parameters highlight the need for a nuanced understanding of the specific interactions between the components of the MEC and their effects on the coated nanoliposome stability. Further mechanistic insights into these interactions will enhance our understanding of nanoliposome behavior and facilitate the development of more stable and efficient nanocarrier systems for various applications.

### 4.4. Proximate Analysis

The proximate composition of the analyzed cheeses reveals statistically significant differences (*p* < 0.05) among the studied samples. The uncoated control cheese (FC) exhibits a high moisture content (57.81% ± 0.40), characteristic of its fresh and unripened nature, retaining a higher water content during its manufacturing process [69]. In contrast, GC displays the lowest moisture content (47.92% ± 0.24). This lower moisture content is likely due to the different properties of rennet curds from goat’s milk compared to cow’s milk used in FC, which typically results in a firmer texture [70]. The specific ripening times for the commercial cheese samples should also be considered, as they also influence moisture content and texture. For instance, the commercial GC typically undergoes a ripening period of approximately 4 to 6 months, compared to the FC, which does not undergo a significant ripening process [71].

Regarding protein content, GC shows the highest concentration (22.17% ± 0.22), attributable to the higher proportion of proteins typically found in goat milk compared to the cow’s milk used in FC [72]. The coated cheese FC-NT/HPMC also exhibits a significant (*p* < 0.05) protein content (19.82% ± 0.32), potentially linked to the coating and the components used in its production.

Goat cheese stands out for its high lipid content (25.17% ± 0.77), characteristic of goat cheeses rich in fat, contributing to its creamy texture and flavor [73,74]. The coated cheese FC-NT/KC also shows a significant (*p* < 0.05) lipid content (4.61% ± 0.07), possibly influenced by grape seed tannins and the properties of kappa carrageenan in retaining fat in the cheese.

The ash content in cheese is related to the presence of minerals and inorganic compounds. Both FC and GC display a notable ash content, possibly due to the mineral composition of the milk used. In contrast, the coated cheeses FC-NT/HPMC and FC-NT/KC show lower ash levels. The reduction in ash content could be due to the interaction between the coating materials and the mineral components in the cheese. The polysaccharides in the coatings might bind to certain minerals, thereby reducing their availability or solubility, which can lead to lower ash content in the final product [75].

Furthermore, the cheese substitute (VC) stands out for its high carbohydrate content (33.98% ± 0.37), attributable to the use of plant-based ingredients instead of dairy [76]. These variations in the proximate composition of the cheeses indicate how the nature of ingredients and manufacturing processes influence the composition and, consequently, the physical properties of cheeses.

The introduction of MEC made with NT and polysaccharides such as HPMC and KC seems to have variable effects on the composition, suggesting the need for further research to understand better its influence on the quality and nutritional value of cheeses.

### 4.5. Biaxial Behavior of Cheeses Coated with MEC

The results provide a detailed insight into how various factors influence the texture of the biaxially compressed cheese samples. Firstly, the apparent influence of the type of coating is highlighted, where the samples coated with NT-HPMC required a significantly higher deformation force compared to those coated with NT-KC [77,78]. This discrepancy is attributed to the intrinsic properties of the polysaccharides used in the coatings, showing that HPMC tends to generate more rigid coatings in contrast to KC, which provides more elastic coatings. Additionally, carrageenan in the coatings appears to accelerate the deformation rate, possibly due to its gelling properties and water-retention capacity [79].

Another finding relates to the coating application method, where the samples coated by immersion require a higher deformation force than those coated by spraying [80]. This difference is mainly attributed to the coating thickness, as immersion results in a thicker layer that demands additional force for deformation. In this regard, the application method emerges as a critical factor in the rheological behavior of the samples.

Compared to laboratory-made cheeses, A significant difference is observed in the biaxial extensional stress between the commercial cheeses GC and VC [81,82]. This disparity could result from variations in the dairy and non-dairy matrices used, the production process, and the days of maturation before acquiring commercial samples.

Analyzing the biaxial extensional viscosity of coated and control cheeses reveals a pseudoplastic behavior in several samples, suggesting a decrease in viscosity as Hencky’s deformation rate increases [20]. This behavior is attributed to the coatings’ structure and composition, which are more pronounced in the samples coated by immersion. The observed differences between the immersion and spray-coated samples are associated with the uniformity of the coating and the formation of a stable biopolymer network [83].

Furthermore, the commercial cheeses GC and VC exhibit higher biaxial extensional viscosity, which is attributed to the proximal composition of the samples. Higher lipid and carbohydrate contents provide structure to the food, requiring more effort for deformation.

The analysis of compression stress highlights the unique response of each cheese type. The control FC shows a slope of 862.43 [Pa/s], indicating a relatively quick response to compression [84]. Moreover, the high coefficient of determination (R^2^) of 0.962 suggests an excellent fit quality of the linear model. This could be related to the typical soft and moist texture of this type of cheese, which deforms and yields to compression more quickly.

On the other hand, GC exhibits the highest slope, with a value of 1385.8 [Pa/s] and an R^2^ of 0.995; this indicates a swift response to compression and excellent model fit quality. Due to their prolonged maturation process, the dense and firm texture of goat cheeses could be related to this rapid response, as the cheese effectively resists compression [85]. VC shows a similar behavior, suggesting a quick response and a good model fit. The texture of vegan cheeses generally resembles that of harder cheeses, which could explain this fast response to compression [86].

The cheeses coated by immersion with NT and polysaccharides FC-NT/HPMC-I and FC-NT/KC-I also show considerable slopes of 685.21 and 769.06 [Pa/s], respectively. These results suggest that nanoliposome and tannin coatings can influence the mechanical response of cheeses, possibly related to the interaction of these ingredients in the cheese matrix [22]. In the case of the cheeses coated by spray with NT and polysaccharides FC-NT-HPMC-A and FC-NT-KC-A, the slopes indicate even faster responses. These results could be related to the specific properties of nanoliposomes and KC, affecting coated cheeses’ texture and compression response. The results suggest that immersion application may moderate the mechanical response of cheese; on the contrary, spraying presents a faster mechanical response, thus providing the cheese with a coating texture that resists compression more firmly [87].

Overall, these findings underscore the importance of carefully considering the composition of coatings, the type of carrageenan, the coating application method, and the type of cheese in the formulation of food products. A deeper understanding of texture is essential for developing food products with desired properties and ensuring the product’s quality. These findings provide valuable guidelines for optimizing formulations and processes in the food industry.

### 4.6. Quality Parameters in Fresh Cheese

The samples coated with HPMC, either by immersion (FC-NT-HPMC-I) or spraying (FC-NT-HPMC-A), exhibit significantly less weight loss compared to the uncoated control (FC). However, it is observed that immersion results in slightly higher weight loss than spraying, which may be due to increased moisture retention in the spray-coated samples [88]. The low weight loss values in both the NT-HPMC-coated samples suggest that applying NT-HPMC provides consistency in moisture retention over time. Similarly, samples coated with KC, either by immersion (FC-NT-KC-I) or spraying (FC-NT-KC-A), also experience less weight loss compared to the control cheese (FC). The choice of application method again influences the magnitude of weight loss, with immersion resulting in slightly higher loss than spraying.

Lastly, the samples from commercial cheeses (GC and VC) exhibited weight loss over the six days. However, this weight loss is significantly lower than the control cheese (FC). This difference could be related to variations in commercial cheeses’ proximal composition and manufacturing process compared to FC.

In summary, these findings underscore the effectiveness of coatings with NT-HPMC and NT-KC in reducing weight loss compared to the uncoated cheese [89].

In the case of FC, a progressive increase in the peroxide index is observed over time. This increase suggests that lipids present in FC are undergoing an oxidation process. The variability in peroxide values, indicated by the rising standard deviation, suggests that oxidation may vary between individual samples, possibly due to differences in lipid quantity, oxygen levels, or cheese storage conditions [90].

NT-HPMC and NT-KC coatings, applied by immersion and spraying, clearly demonstrate antioxidant effects in reducing peroxide formation in cheese during storage. This antioxidant effect is attributed to the ability of these coatings to act as physical barriers, limiting the exposure of cheese lipids to environmental oxygen and the presence of tannins, which are gradually released from the coating [91]. The cheese coated with NT-HPMC stands out for maintaining consistent antioxidant protection over time, suggesting constant effectiveness. On the other hand, NT-KC is also effective but shows more significant variability in peroxide values and standard deviations, which could be due to specific sample factors. The choice between NT-HPMC and NT-KC may depend on the desired consistency in antioxidant protection and other specific factors related to the product or manufacturing process.

Furthermore, the samples from commercial cheeses GC and VC also exhibit lower peroxide indices than FC, suggesting that these commercial cheeses have been formulated or processed to reduce peroxide formation. This reduction could be attributed to selecting ingredients (additives) and manufacturing methods that minimize exposure of cheese lipids to oxygen. The low variability in peroxide values and lower standard deviations indicate that these commercial cheeses maintain consistent quality and are less prone to oxidation.

## 5. Conclusions

During the analysis of the proximal composition, significant differences (*p* < 0.05) were observed in the contents of moisture, proteins, lipids, ash, and carbohydrates between cheeses coated with nanoliposomes (FC-NT/HPMC and FC-NT/KC) and commercial cheeses (GC and VC). Particularly noteworthy is the significantly high protein content of 19.82% ± 0.32 in the FC-NT/HPMC coated cheese, indicating a potential influence of the coating and its components on its production.

The analysis of biaxial behavior revealed that coating, application method, and cheese type significantly influenced texture and compression response, with biaxial extensional viscosity values ranging from 1036 to 1083 [Pa·s]. Furthermore, MEC exhibited antioxidant properties by reducing peroxide formation by 75% compared to the uncoated cheese, limiting weight loss to below 5% during refrigerated storage.

In terms of quality parameters, the nanoliposome coatings (NT-HPMC and NT-KC) proved effective in significantly reducing (*p* < 0.05) weight loss and acted as antioxidant barriers, limiting peroxide formation during storage. This antioxidant effect was attributed to the ability of these coatings to act as physical barriers and the progressive release of tannins. The commercial cheeses also showed less susceptibility to oxidation, emphasizing the importance of formulation and processing in the final product’s quality.

These results underscore the complexity of the interaction between coatings, cheese composition, and processing methods, emphasizing the need to carefully consider these factors to develop food products with desired properties and ensure quality.

## Figures and Tables

**Table 1:** Multi-level factorial design *.

Factors	Low	High	Levels	Units	Answer	Units
Tween-80	1	1	3	[%]	ρ	[kg/m^3^]
GLY	5	5	3	[%]	Υ	[mN/m]
					OD	[Dimensionless]

* Software STATGRAPHICS Centurion XVI (v.16.1.03), 2022. GLY: glycerol; ρ: density; Υ: surface tension; OD: optical density.

**Table 2:** Stability study of NT during the refrigerated storage period *.

Days	MPS [nm]	PDI	Diffusion Coefficient [µm^2^/s]	Z-Potential [mV]	Conductivity [mS/cm]	Transmittance [%]
0	450 ± 3 ^d^	0.299 ± 0.10 ^d^	1.088 ± 0.04 ^a^	30.47 ± 1.89 ^b^	2.98 ± 0.04 ^c^	0.069 ± 0.01 ^a^
1	434 ± 2 ^c^	0.248 ± 0.04 ^b^	1.129 ± 0.05 ^a^	25.58 ± 2.65 ^a^	2.94 ± 0.09 ^c^	0.071 ± 0.02 ^a^
2	356 ± 5 ^c^	0.260 ± 0.05 ^c^	1.375 ± 0.04 ^b^	36.31 ± 2.32 ^b^	2.83 ± 0.08 ^bc^	0.066 ± 0.03 ^a^
3	337 ± 3 ^b^	0.265 ± 0.04 ^c^	1.453 ± 0.07 ^c^	29.38 ± 3.12 ^ab^	2.62 ± 0.02 ^b^	0.071 ± 0.01 ^a^
4	349 ± 4 ^c^	0.238 ± 0.09 ^ab^	1.405 ± 0.05 ^c^	42.66 ± 6.57 ^c^	2.61 ± 0.07 ^b^	0.071 ± 0.02 ^a^
5	345 ± 3 ^c^	0.226 ± 0.09 ^a^	1.420 ± 0.02 ^c^	40.05 ± 2.62 ^c^	2.62 ± 0.09 ^b^	0.069 ± 0.02 ^a^
6	331 ± 5 ^a^	0.259 ± 0.03 ^c^	1.479 ± 0.06 ^c^	28.59 ± 3.03 ^ab^	1.84 ± 0.06 ^a^	0.067 ± 0.04 ^a^
7	319 ± 3 ^a^	0.218 ± 0.05 ^a^	1.537 ± 0.06 ^d^	47.89 ± 5.23 ^c^	2.86 ± 0.03 ^bc^	0.062 ± 0.04 ^a^
8	320 ± 4 ^a^	0.216 ± 0.08 ^a^	1.528 ± 0.01 ^d^	27.93 ± 1.02 ^ab^	2.61 ± 0.07 ^b^	0.065 ± 0.01 ^a^
9	324 ± 3 ^a^	0.254 ± 0.02 ^c^	1.511 ± 0.02 ^d^	27.86 ± 1.14 ^ab^	3.01 ± 0.03 ^c^	0.066 ± 0.03 ^a^
10	329 ± 4 ^a^	0.263 ± 0.06 ^c^	1.227 ± 0.09 ^a^	28.57 ± 0.65 ^ab^	2.69 ± 0.07 ^b^	0.063 ± 0.03 ^a^
11	321 ± 3 ^a^	0.220 ± 0.04 ^a^	1.528 ± 0.09 ^d^	38.03 ± 1.72 ^a^	2.74 ± 0.09 ^b^	0.065 ± 0.02 ^a^
12	324 ± 3 ^a^	0.240 ± 0.06 ^b^	1.432 ± 0.02 ^c^	30.19 ± 0.29 ^ab^	3.02 ± 0.02 ^c^	0.063 ± 0.02 ^a^
13	327 ± 2 ^a^	0.243 ± 0.04 ^b^	1.451 ± 0.04 ^c^	32.65 ± 2.46 ^b^	3.02 ± 0.01 ^c^	0.064 ± 0.01 ^a^
14	329 ± 3 ^a^	0.241 ± 0.03 ^b^	1.585 ± 0.09 ^d^	34.74 ± 1.62 ^b^	2.99 ± 0.03 ^c^	0.064 ± 0.01 ^a^
15	332 ± 2 ^a^	0.242 ± 0.05 ^b^	1.476 ± 0.02 ^c^	37.96 ± 0.08 ^c^	3.00 ± 0.03 ^c^	0.063 ± 0.02 ^a^

* Results are presented as means ± standard deviation. Different letters in the same column indicate significant differences (*p* < 0.05). MPS: mean particle size; PDI: polydispersity index.

**Table 3:** Proximate composition of coated cheeses with MEC and studied commercial cheeses *.

Sample	Moisture [%]	Protein [%]	Lipids [%]	Ashes [%]	Carbohydrates [%]
FC	57.8 ± 0.4 ^d^	17.9 ± 1.3 ^c^	3.6 ± 0.2 ^ab^	3.1 ± 0.1 ^c^	17.5 ± 1.5 ^b^
FC-NT/HPMC	54.9 ± 0.3 ^c^	19.8 ± 0.3 ^d^	3.0 ± 0.3 ^a^	2.8 ± 0.1 ^b^	19.4 ± 0.4 ^b^
FC-NT/KC	54.2 ± 0.3 ^c^	16.1 ± 0.2 ^b^	4.6 ± 0.1 ^b^	2.6 ± 0.1 ^a^	22.4 ± 0.2 ^c^
GC	47.9 ± 0.2 ^a^	22.2 ± 0.2 ^e^	25.2 ± 0.8 ^d^	3.1 ± 0.1 ^c^	1.7 ± 0.8 ^a^
VC	49.3 ± 0.4 ^b^	2.1 ± 0.1 ^a^	7.4 ± 0.3 ^c^	7.2 ± 0.1 ^d^	33.9 ± 0.4 ^d^

* Results are presented as means ± standard deviation. Different letters in the same column indicate significant differences (*p* < 0.05). FC: control fresh cheese; FC-NT/HPMC: fresh cheese coated with nanoliposomes encapsulating grape seed tannins and hydroxypropyl methylcellulose; FC-NT/KC: fresh cheese coated with nanoliposomes encapsulating grape seed tannins and kappa carrageenan; GC: goat cheese; VC: vegan cheese substitute.

**Table 4:** Fitting results of biaxial behavior curves of cheese samples *.

Sample	Velocity [mm/s]	A [Pa/s]	R^2^	t_0_ [s]	θ (t − t_0_) < t_R_ [s]
FC	1	862.43	0.962	0.02	θ < (8.32 − t_0_)
FC-NT-HPMC-I	1	685.21	0.958	0.02	θ < (8.40 − t_0_)
FC-NT-HPMC-A	1	1036.8	0.919	0.02	θ < (8.10 − t_0_)
FC-NT-KC-I	1	769.06	0.941	0.02	θ < (8.45 − t_0_)
FC-NT-KC-A	1	1083.2	0.895	0.02	θ < (8.50 − t_0_)
GC	1	1385.8	0.995	0.02	θ < (5.96 − t_0_)
VC	1	1664.8	0.946	0.02	θ < (7.28 − t_0_)

* Results are presented as means ± standard deviation. FC: control fresh cheese; FC-NT-HPMC: fresh cheese coated with nanoliposomes that encapsulate grape seed tannins and hydroxypropylmethylcellulose; FC-NT-KC: fresh cheese coated with nanoliposomes that encapsulate grape seed tannins and kappa carrageenan; GC: goat cheese; VC: vegan cheese substitute.

**Table 5:** Peroxide index expressed in Meq. O_2_/kg of cheese in a storage period under accelerated conditions *.

	Days						
Sample	0	1	2	3	4	5	6
FC	2.2 ± 0.2 ^b^	3.2 ± 0.2 ^b^	3.7 ± 0.3 ^b^	5.1 ± 0.4 ^b^	5.7 ± 0.4 ^c^	6.5 ± 0.2 ^c^	9.1 ± 0.4 ^c^
FC-NT-HPMC-I	0.7 ± 0.3 ^a^	0.8 ± 0.2 ^a^	1.4 ± 0.2 ^a^	1.7 ± 0.3 ^a^	1.9 ± 0.2 ^a^	1.8 ± 0.5 ^a^	2.8 ± 0.5 ^ab^
FC-NT-HPMC-A	0.8 ± 0.5 ^a^	1.4 ± 0.2 ^a^	1.6 ± 0.2 ^a^	1.7 ± 0.3 ^a^	1.8 ± 0.2 ^a^	1.9 ± 0.4 ^a^	2.2 ± 0.4 ^a^
FC-NT-KC-I	1.0 ± 0.3 ^a^	1.4 ± 0.5 ^a^	1.8 ± 0.2 ^a^	2.0 ± 0.3 ^a^	2.1 ± 0.2 ^a^	3.0 ± 0.3 ^b^	3.1 ± 0.5 ^ab^
FC-NT-KC-A	1.2 ± 0.2 ^ab^	1.3 ± 0.3 ^a^	1.9 ± 0.4 ^a^	2.1 ± 0.2 ^a^	2.3 ± 0.3 ^a^	3.2 ± 0.4 ^b^	3.9 ± 0.6 ^b^
GC	2.2 ± 0.4 ^b^	3.0 ± 0.3 ^b^	3.4 ± 0.2 ^b^	4.2 ± 0.5 ^b^	4.7 ± 0.3 ^b^	6.2 ± 0.2 ^c^	9.4 ± 0.4 ^c^
VC	2.1 ± 0.5 ^b^	3.0 ± 0.3 ^b^	3.7 ± 0.3 ^b^	4.8 ± 0.4 ^b^	6.9 ± 0.2 ^d^	7.9 ± 0.5 ^d^	9.7 ± 0.3 ^c^

* Results are presented as means ± standard deviation. Different letters in the same column indicate significant differences (*p* < 0.05). FC: control fresh cheese; FC-NT-HPMC: fresh cheese coated with nanoliposomes that encapsulate grape seed tannins and hydroxypropylmethylcellulose; FC-NT-KC: fresh cheese coated with nanoliposomes that encapsulate grape seed tannins and kappa carrageenan; GC: goat cheese; VC: vegan cheese substitute.

## Data Availability

The data presented in this study are available on request from the corresponding author. The data are not publicly available because it belongs to an ongoing project.
